# Central nervous system involvement in childhood acute lymphoblastic leukemia is linked to upregulation of cholesterol biosynthetic pathways

**DOI:** 10.1038/s41375-022-01722-x

**Published:** 2022-10-26

**Authors:** A. Cousins, O. Olivares, E. Markert, A. Manoharan, X. Bubnova, S. Bresolin, M. Degn, Z. Li, D. Silvestri, G. McGregor, S. Tumanov, D. Sumpton, J. J. Kamphorst, A. M. Michie, P. Herzyk, M. G. Valsecchi, A. E. Yeoh, K. Schmiegelow, G. te Kronnie, E. Gottlieb, C. Halsey

**Affiliations:** 1 Wolfson Wohl Cancer Research Centre, School of Cancer Sciences, College of Medical Veterinary and Life Sciences, University of Glasgow, Glasgow, UK; 2 Cancer Research UK Beatson Institute, Glasgow, UK; 3 Department of Women’s and Children’s Health, University of Padova, Padova, Italy; 4 Department of Pediatrics and Adolescent Medicine, The Juliane Marie Centre, The University Hospital Rigshospitalet, Copenhagen, Denmark; 5 VIVA-NUS Centre for Translational Research in Acute Leukaemia, Department of Paediatrics, Yong Loo Lin School of Medicine, National University of Singapore, Singapore, 117599 Singapore; 6 Center of Biostatistics for Clinical Epidemiology, Department of Health Science, University of Milano-Bicocca, Milano, Italy; 7 Paul O’Gorman Leukaemia Research Centre, School of Cancer Sciences, College of Medical Veterinary and Life Sciences, University of Glasgow, Glasgow, UK; 8 Glasgow Polyomics, College of Medical Veterinary and Life Sciences, University of Glasgow, Glasgow, UK; 9 Institute of Molecular, Cell and Systems Biology, College of Medical Veterinary and Life Sciences, University of Glasgow, Glasgow, UK; 10 VIVA-University Children’s Cancer Centre, Khoo Teck Puat-National University Children’s Medical Institute, National University Hospital, National University Health System, Singapore, 119228 Singapore; 11 Institute of Clinical Medicine, Faculty of Medicine, University of Copenhagen and Juliane Marie Centre, the University Hospital Rigshospitalet, Copenhagen, Denmark; 12 Department of Molecular Medicine, University of Padova, Padova, Italy; 13 The Ruth and Bruce Rappaport Faculty of Medicine, Technion - Israel Institute of Technology, Haifa, Israel

**Keywords:** Translational research, Acute lymphocytic leukaemia, Cancer metabolism

Treatment for childhood acute lymphoblastic leukemia (ALL) requires intensive intrathecal and systemic therapy to reduce the risk of central nervous system (CNS) relapse. This treatment is associated with high rates of neurotoxicity as well as being onerous for both patients and healthcare systems [1]. Improving CNS-directed therapy, requires understanding of the mechanisms of leukemic survival in this microenvironment, accompanied by better prognostic and predictive biomarkers for CNS leukemia. The former will underpin discovery of treatments with reduced toxicity, the latter is essential for the development of risk-adapted CNS-directed therapy.

Leukemic cells in the CNS reside within the leptomeninges, between the arachnoid and pial stromal layers, bathed in cerebrospinal fluid (CSF). CSF is relatively nutrient poor compared to plasma with low levels of protein, lipids, glucose, and oxygen. This is in marked contrast to the highly cellular and relatively nutrient-rich environment in the bone marrow (BM), spleen, and other peripheral organs where ALL cells accumulate. One particular difference between the CNS and periphery is in the bioavailability of cholesterol. Cholesterol is relatively abundant in the CNS as a whole – mainly found in myelinated neural sheaths in the brain parenchyma—but is extremely scarce in the CSF [2]. The cholesterol in the CNS consists of a specific isoform (24(S)-hydroxycholesterol) which is produced only in the CNS, mainly by glial neuron-supporting cells, with no “systemic” cholesterol detected [3]. In addition, the lipoproteins which normally transport cholesterol within the systemic circulation are not found in the CNS, with CNS-specific lipoproteins detected in CSF [3]. Cholesterol and its intermediates are of vital importance to cell maintenance, survival and growth and cholesterol biosynthesis is increasingly recognised as a key pathway perturbed in cancer biology [4].

We hypothesised that leukemic cells in the CNS undergo metabolic adaptations to their unique microenvironment that might be therapeutically exploitable. Here we show that the cholesterol biosynthetic pathway is highly upregulated in CNS leukemia cells and that inhibition of the pathway results in reduced cell viability and increased apoptosis in vitro. We provide pre-clinical evidence that active CNS leukemia is associated with reduction in CSF cholesterol levels, and that an upregulated cholesterol synthesis gene-signature may identify patients at increased risk of leukemic relapse, particularly CNS relapse.

To investigate transcriptional adaptations to the CNS microenvironment, we retrieved leukemic cells from the CNS and spleen of NSG (NOD-scid IL2Rg^null^) mice xenotransplanted with two human B-cell precursor (BCP) leukemia cell lines—SEM and REH (full details in Supplementary materials and methods). Transcripts were initially aligned against the murine genome to remove signatures from any contaminating murine cells, prior to gene set enrichment analysis. The low-density lipoprotein receptor (LDLR)—the main receptor governing cholesterol uptake - and genes of the cholesterol biosynthesis pathway were amongst the most highly upregulated genes/processes in CNS-derived leukemia cells relative to spleen-derived leukemia cells (Fig. 1a, Supplementary Fig. 1). To further explore this association, we examined publicly available human datasets [5] which confirmed significant upregulation of cholesterol homeostasis and biosynthesis genes in CNS-derived blasts from patients with ALL (Fig. 1b).

**Fig. 1:**
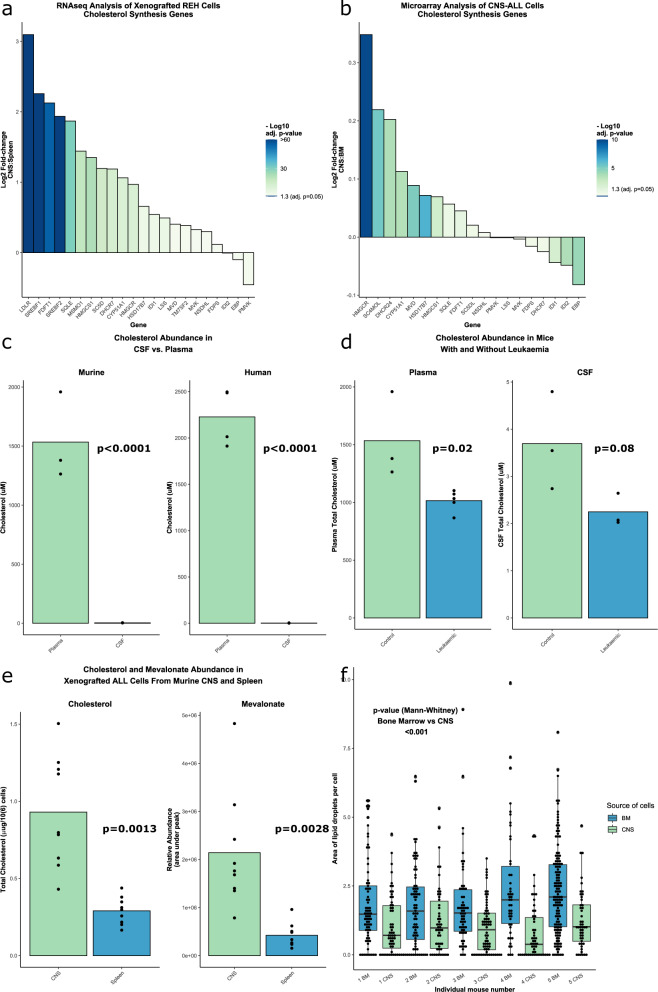
Cholesterol biosynthesis is upregulated in BCP-ALL cells in the CNS. **a** Waterfall plot showing differential expression of the genes in the cholesterol biosynthesis pathway between xenografted REH (BCP-ALL cell line) cells retrieved from the CNS and the spleen of NSG mice. *n* = 3 experiments (3-4 mice per experiment). **b** Waterfall plot showing differential expression of the genes in the cholesterol biosynthesis pathway between human BCP-ALL cells retrieved from the CSF of paediatric patients at CNS relapse vs BM at diagnosis or BM relapse [5]. **a**, **b** Adjusted *p*-values calculated using Benjamini-Hochberg method. **c** Bar charts showing cholesterol abundance in plasma and CSF of normal control NSG mice and humans. Mice: *n* = 3 mice (both groups); Human: *n* = 4 (plasma), *n* = 5 (CSF) **d** Bar charts showing cholesterol abundance in plasma and CSF between NSG mice with and without leukemia. Plasma: *n* = 3 (control), 5 (leukemic); CSF: *n* = 3 (both groups) **e** Bar charts showing intracellular cholesterol and mevalonate abundance in xenografted SEM cells retrieved from the CNS and spleen of NSG mice. n = 9 mice (all groups). **d**–**f** *P*-values calculated using 2-tailed student’s *t*-test. **f** Boxplot showing area of lipid droplets in xenografted SEM cells retrieved from CNS and bone marrow of NSG mice. *n* = 658 cells (385 bone marrow/273 CNS) analysed from 5 mice. *P*-value calculated using Wilcoxon rank-sum/Mann-Whitney U test.

We were able to confirm the very low levels of cholesterol in CSF from mice and humans (Fig. 1c) and show that the presence of active leukemia is associated with a trend towards further reduction of these levels (Fig. 1d, Supplementary Fig. 2a). Reduction in cholesterol levels may be due to active uptake by CNS leukemia cells from the CSF. In support of this, measurement of total cholesterol and the cholesterol intermediate mevalonate showed increased levels of these metabolites in SEM cells retrieved from the CNS compared to those from the spleen (Fig. 1e). There were reduced lipid droplets in SEM cells retrieved from the CNS compared with those from the bone marrow (Fig. 1f).

To investigate whether cholesterol synthesis is important for ALL cell survival we treated SEM cells in vitro with the HMGCoA reductase inhibitor Simvastatin. This led to a reduction in cell viability with an increase in apoptosis (Supplementary Fig. 2b). On-target effects were confirmed by rescue of the phenotype with mevalonate which is the downstream product of HMGCoA reductase (Supplementary Fig. 2b). Interestingly supplementation with cholesterol was unable to rescue the phenotype (Supplementary Fig. 2c), suggesting that upstream intermediates between mevalonate and cholesterol are required by leukemia cells. In common with other groups [6] we were unable to identify an in vivo reduction in leukemia burden in xenografted mice treated with simvastatin (Supplementary Fig. 2d, e). SEM cells with genetic knockdown/knockout of the HMGCR gene were found to be not viable in-vitro (data not shown).

Finally, we investigated whether an upregulated cholesterol synthesis signature in ALL cells from diagnostic BM samples was associated with an increased risk of leukemia relapse. Publicly available data for children with high-risk ALL treated on the Children’s Oncology Group (COG) P9906 trial were obtained from the US National Cancer Institute TARGET phase 1 ALL project (http://ocg.cancer.gov/programs/target) and NCBI Geo DataSets GSE GSE11877. Using a panel of 21 cholesterol pathway genes (see supplementary methods for details), we found that upregulated expression (at least 2 genes upregulated with a Z score > 1.5) was associated with the risk of isolated CNS relapse (Fig. 2a, b), and reduced overall survival (Supplementary Fig. 3b, d), but not BM relapse (Supplementary Fig. 3a, c). Additionally, cholesterol gene upregulation (Z-score > 1.2) increased CNS relapse risk in a dose-dependent manner (Fig. 2c). This increased CNS relapse risk was independent of minimal residual disease (MRD) risk group and in this analysis, upregulation of cholesterol synthesis genes was more predictive of CNS relapse than day 28 MRD levels (Fig. 2b). In contrast, MRD remained the most relevant risk factor for BM relapse and overall survival (Supplementary Fig. 3a–d). The P9906 trial recruited high-risk patients [7] and consequently had relatively high rates of CNS relapse. Examination of additional international cohorts with available gene expression profiling confirmed the association of upregulated cholesterol biosynthesis with leukemia relapse (Fig. 2d–f), but did not have sufficient power to validate an independent effect on CNS relapse rates (Supplementary Fig. 3e, g, i). There was a trend towards reduced overall survival, but this only reached statistical significance in 1 out of 3 datasets (Supplementary Fig. 3f, h, j).

**Fig. 2:**
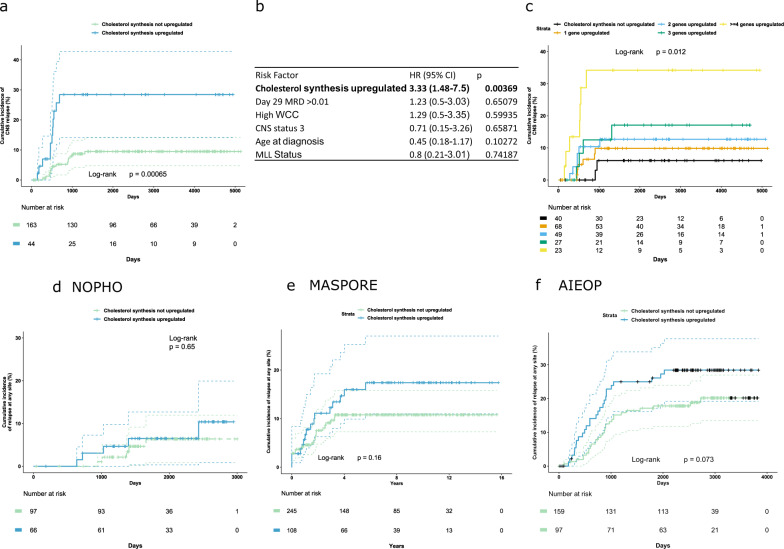
Upregulated cholesterol synthesis at diagnosis is associated with increased risk of isolated CNS relapse. **a** Kaplan-Meier curve showing cumulative incidence of isolated CNS relapse in children with high-risk BCP-ALL by upregulation of cholesterol biosynthesis genes in the TARGET dataset. Dotted lines indicate 95% confidence intervals. **b** Cox Proportional Hazards model of risk of CNS relapse in this dataset with traditional risk factors (MRD positivity of >0.01% at day 29 of induction therapy, WCC > 50 × 10^9^/L, > 5 white cells/μL CSF with ALL blasts identified, Age >10 years, MLL rearrangement identified) and upregulation of cholesterol synthesis genes. **c** Kaplan-Meier curve showing cumulative incidence of isolated CNS relapse with increasing numbers of genes in cholesterol biosynthesis pathway upregulated (defined as z-score for gene expression ≥ 1.2). Cumulative incidence of any relapse by upregulation of cholesterol synthesis in international datasets from the (**d**) NOPHO, (**e**) MASPORE, and (**f**) AIEOP groups.

In this study complementary in vitro, in vivo and primary patient data indicate a consistent association between upregulation of cholesterol metabolism and CNS leukemia. Previous reports have indicated that presentation of acute leukemia can be associated with low plasma/serum cholesterol levels [8], which subsequently rise during treatment [9]. The aetiology of systemic hypocholesterolemia is unproven but may be due to enhanced uptake by leukemia cells, or by an acute inflammation stimulating hepatic uptake [10]. The levels of cholesterol in CSF have never previously been examined in this context, the lack of cholesterol transport from the periphery makes this a particularly attractive choice as an independent biomarker for CNS leukemia. Our data showing a reduction in lipid droplets in ALL cells in the CNS are further confirmation of the importance of metabolic adaptation, in particular lipid metabolism, in CNS ALL as has been described recently [11].

There are several potential reasons why ALL cells may require cholesterol in the CNS microenvironment. A summary of our findings and a proposed model is shown in graphical form in Supplemental Fig. 4. Cholesterol and other sterols are important for membrane integrity and fluidity. In addition, intermediates in the cholesterol synthetic pathway participate in many essential cellular processes. These include ubiquinone (coenzyme Q), involved in electron transport and cell respiration; farnesyl and geranylgeranyl isoprenoids, required for the covalent binding of proteins such as the Ras family to membranes; dolichol, which is needed for glycoprotein synthesis; and isopentenyladenine, essential for certain tRNA function and protein synthesis. It is likely that an increased reliance on one or more of these processes, under conditions of cellular stress, underlies the pathway upregulation observed in CNS ALL. One attractive candidate is the role of the cholesterol biosynthesis pathway in farnesylation of Ras—the Ras/Raf/MEK/ERK pathway is known to be activated in CNS leukemia and particularly important in low nutrient conditions [12, 13]. Use of Selumetinib—a MEK1/2 inhibitor—eradicates CNS leukemia in patient derived xenografts [13]. Another candidate mechanism is regulation of receptor signalling via cholesterol-rich membrane domains—there is experimental evidence that VEGFR stimulation promotes FLT-1 localization in such cholesterol-rich domains in ALL cell lines [14]. Again, enhanced VEGF activity is causally linked to CNS leukemia [15]. The role of cytokine signalling (e.g., TNF signalling) has been explored previously in CNS ALL [16]. There are clearly close links between cell signalling and cell metabolism, and this area merits further study.

The lack of efficacy of statin therapy in vivo may reflect inadequate plasma and CSF drug levels and/or that compensatory pathways may operate in vivo. Our results are similar to those reported by Samuels *et al*, who identified a potential simvastatin vulnerability in drug-resistant ALL. They confirmed good in vitro activity of Simvastatin but a lack of in vivo efficacy, possibly due to low plasma levels achieved with the maximally tolerated dose [6].

CNS leukemia remains a clinical challenge. In particular, the lack of adequate prognostic markers indicating risk of CNS relapse, and the lack of sensitive biomarkers for the presence of CNS infiltration in the leptomeninges hinder the development of risk-adapted CNS-directed therapy. Our findings identify low CSF cholesterol levels—likely due to cellular uptake by leukemia cells - as a potential clinical biomarker for the presence of CNS leukemia. In addition, analysis of patient outcome data indicates that an upregulated cholesterol biosynthetic signature is associated with an increased risk of relapse—particularly in the CNS. Further verification of these potential prognostic biomarkers in large prospective clinical cohorts is now required.

## Supplementary information

Supplemental Figure 1

Supplemental Figure 2

Supplemental Figure 3

Supplemental Figure 4

Supplemental Methods

Supplemental Figure Legends

## Data Availability

The RNA-Seq data from these xenotransplantation experiments are available via accession number GSE135115 under the title “Gene expression profiles of MLL-AF4 and TEL-AML1 acute lymphoblastic leukaemia blasts retrieved from central nervous system and spleen”. This has two datasets, GSE135113 “Gene expression profiles of MLL-AF4 acute lymphoblastic leukemia blasts retrieved from central nervous system and spleen”, and GSE135114 “Gene expression profiles of TEL-AML1 acute lymphoblastic leukemia blasts retrieved from central nervous system and spleen”. Results from this dataset have previously been published [11].
