# Supplementary figures and images for: Central nervous system involvement in childhood acute lymphoblastic leukemia is linked to upregulation of cholesterol biosynthetic pathways
Source: Leukemia. 2022 Oct 26;36(12):2903–7. doi: 10.1038/s41375-022-01722-x (PMC9712090; doi:10.1038/s41375-022-01722-x)

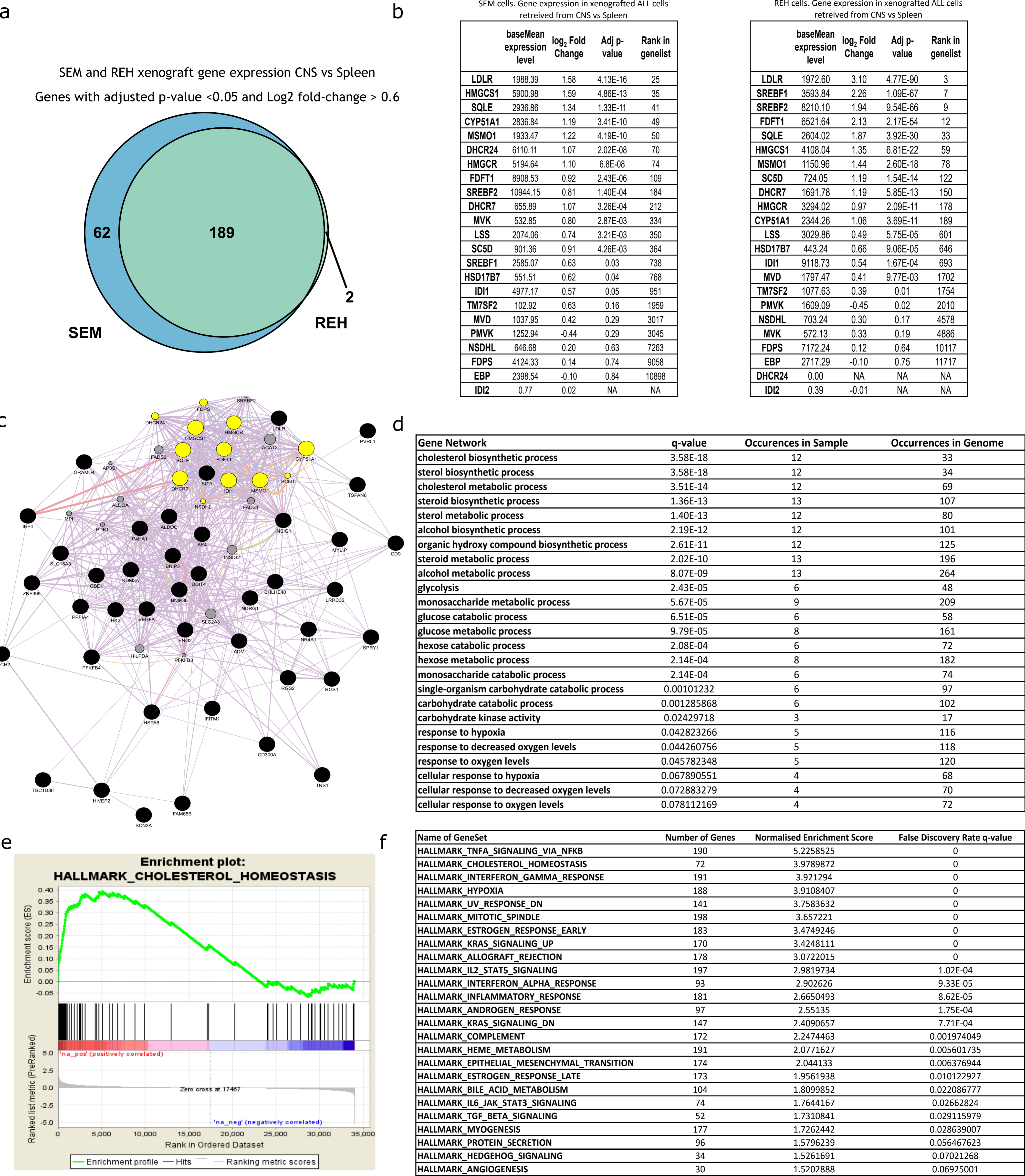

Supplement: Supplementary file 1 — Supplemental Figure 1 [file 41375_2022_1722_MOESM1_ESM.pdf]

## Bone Marrow

## Leptomeninges

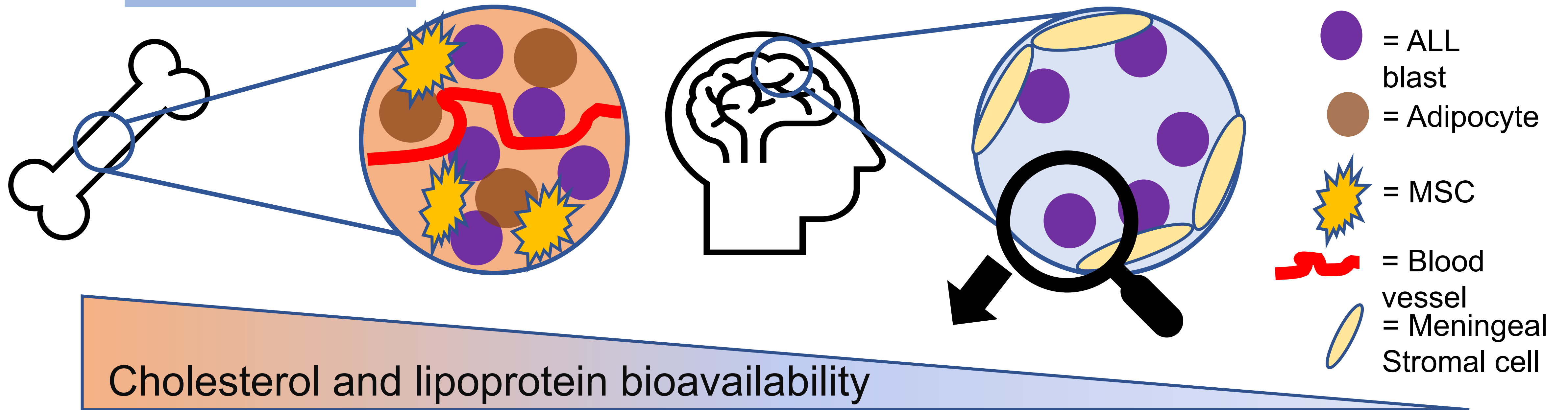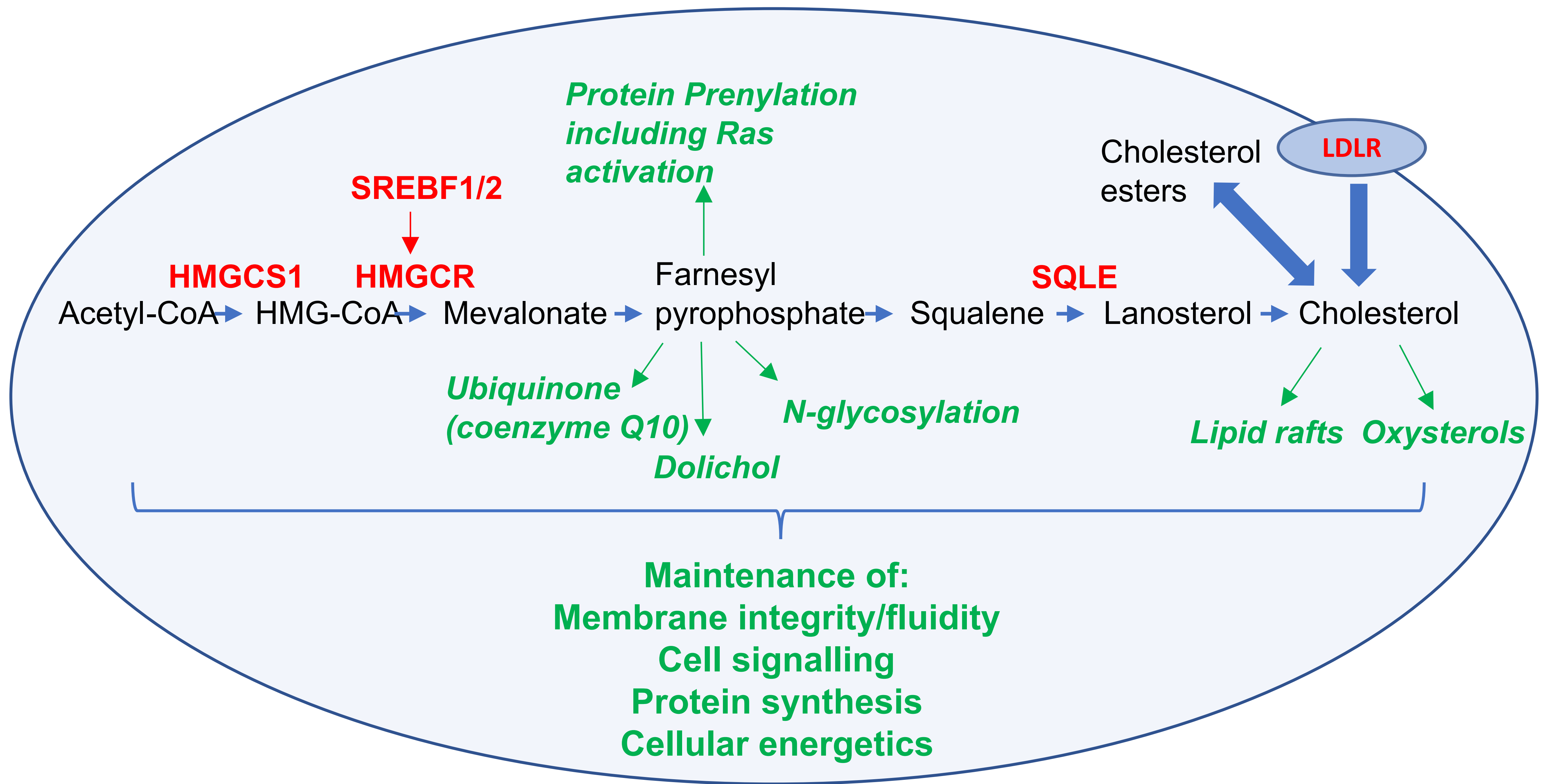

Supplement: Supplementary file 4 — Supplemental Figure 4 [file 41375_2022_1722_MOESM4_ESM.pdf]
