# Supplementary material for: Central nervous system involvement in childhood acute lymphoblastic leukemia is linked to upregulation of cholesterol biosynthetic pathways
Source: Leukemia. 2022 Oct 26;36(12):2903–7. doi: 10.1038/s41375-022-01722-x (PMC9712090; doi:10.1038/s41375-022-01722-x)
Supplement: Supplementary file 2 — Supplemental Figure 2 [file 41375_2022_1722_MOESM2_ESM.pdf]

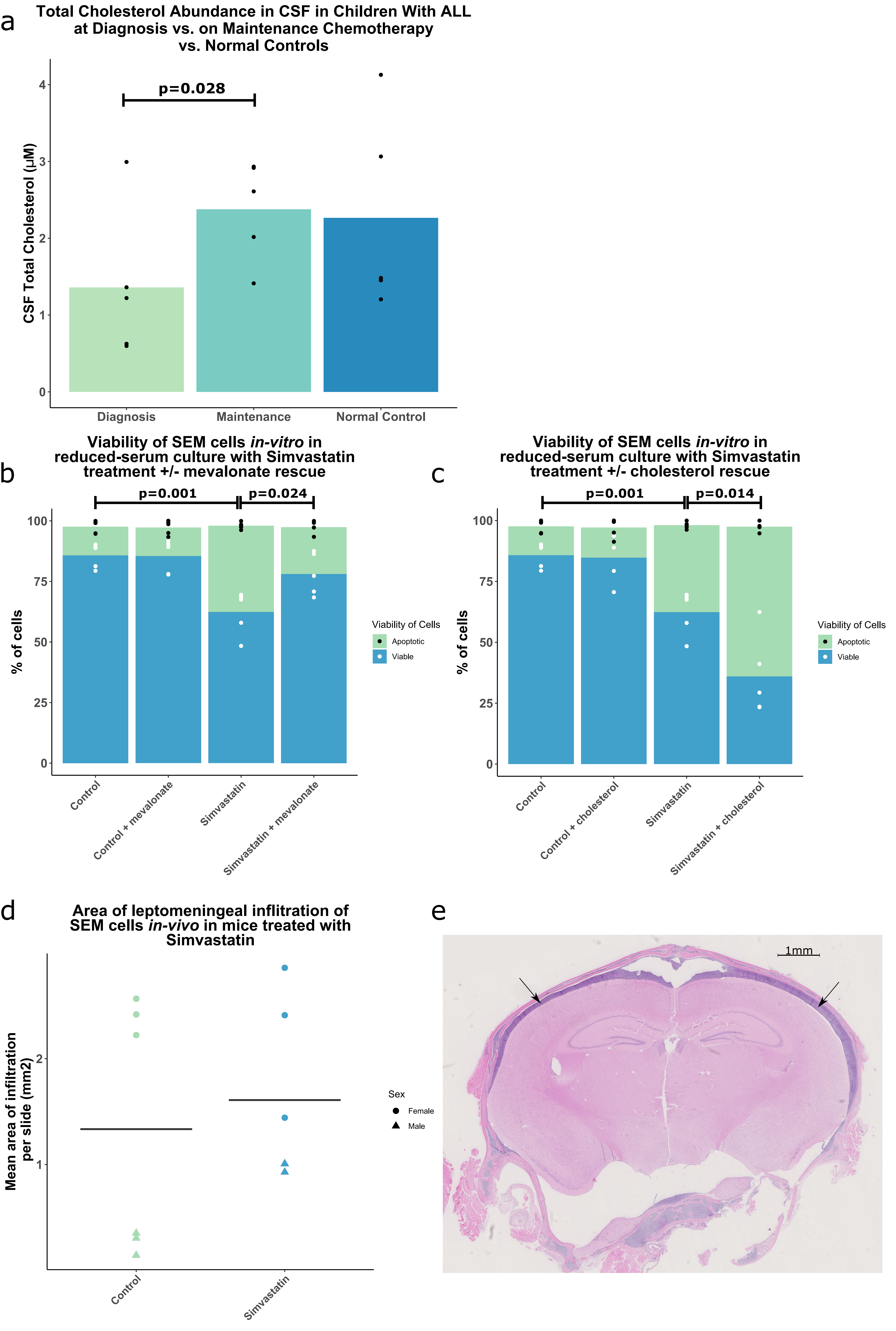
a
Total Cholesterol Abundance in CSF in Children With ALL at Diagnosis vs. on Maintenance Chemotherapy vs. Normal Controls
p=0.028
CSF Total Cholesterol (μM)
0
1
2
3
4
Diagnosis
Maintenance
Normal Control
b
Viability of SEM cells in-vitro in reduced-serum culture with Simvastatin treatment +/- mevalonate rescue
p=0.001
p=0.024
% of cells
0
25
50
75
100
Control
Control + mevalonate
Simvastatin
Simvastatin + mevalonate
Viability of Cells
Apoptotic
Viable
c
Viability of SEM cells in-vitro in reduced-serum culture with Simvastatin treatment +/- cholesterol rescue
p=0.001
p=0.014
% of cells
0
25
50
75
100
Control
Control + cholesterol
Simvastatin
Simvastatin + cholesterol
Viability of Cells
Apoptotic
Viable
d
Area of leptomeningeal inflitration of SEM cells in-vivo in mice treated with Simvastatin
Mean area of infiltration per slide (mm2)
1
2
Control
Simvastatin
Sex
Female
Male
e
1mm
