# Supplementary material for: Central nervous system involvement in childhood acute lymphoblastic leukemia is linked to upregulation of cholesterol biosynthetic pathways
Source: Leukemia. 2022 Oct 26;36(12):2903–7. doi: 10.1038/s41375-022-01722-x (PMC9712090; doi:10.1038/s41375-022-01722-x)

a

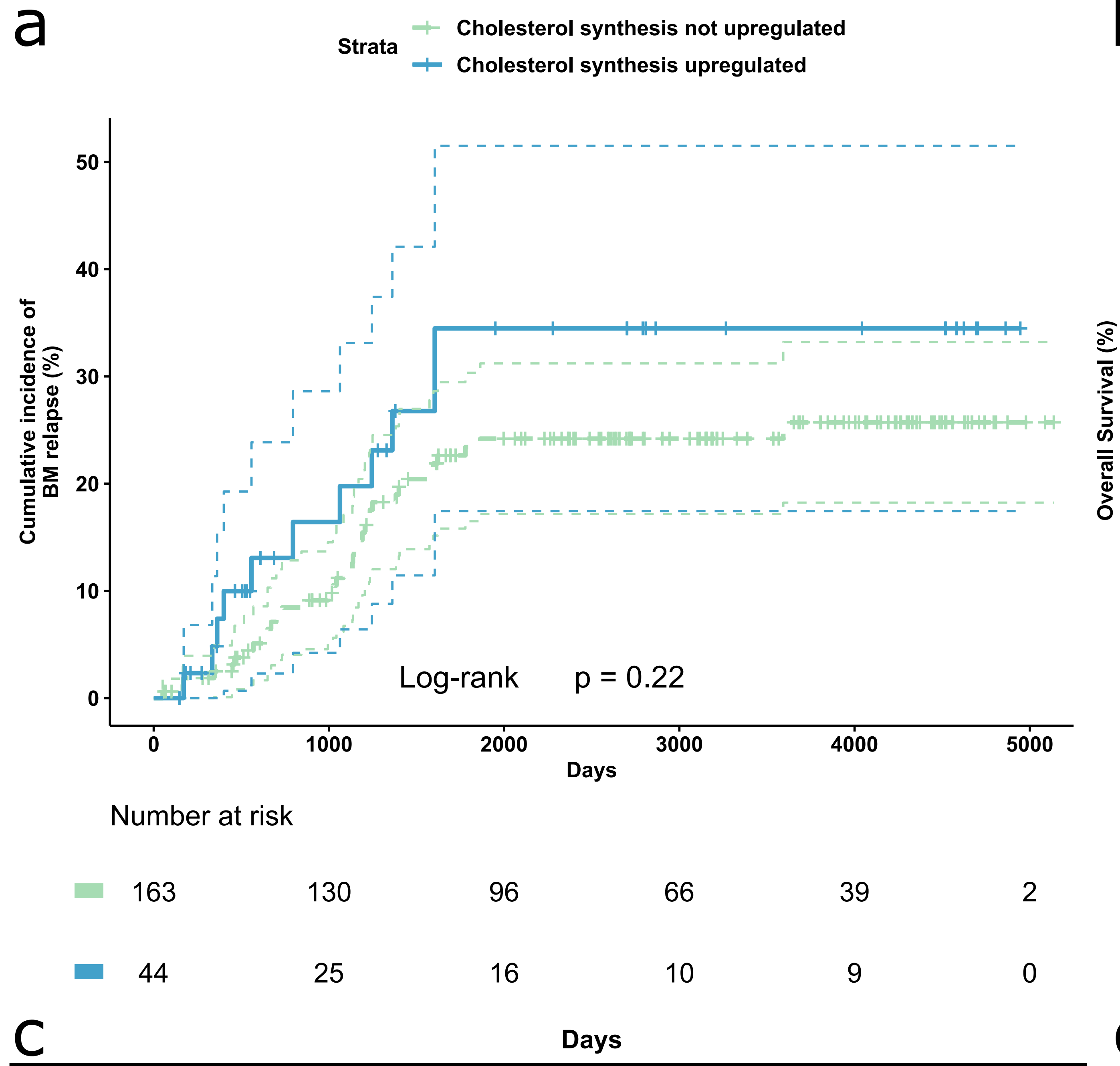

b

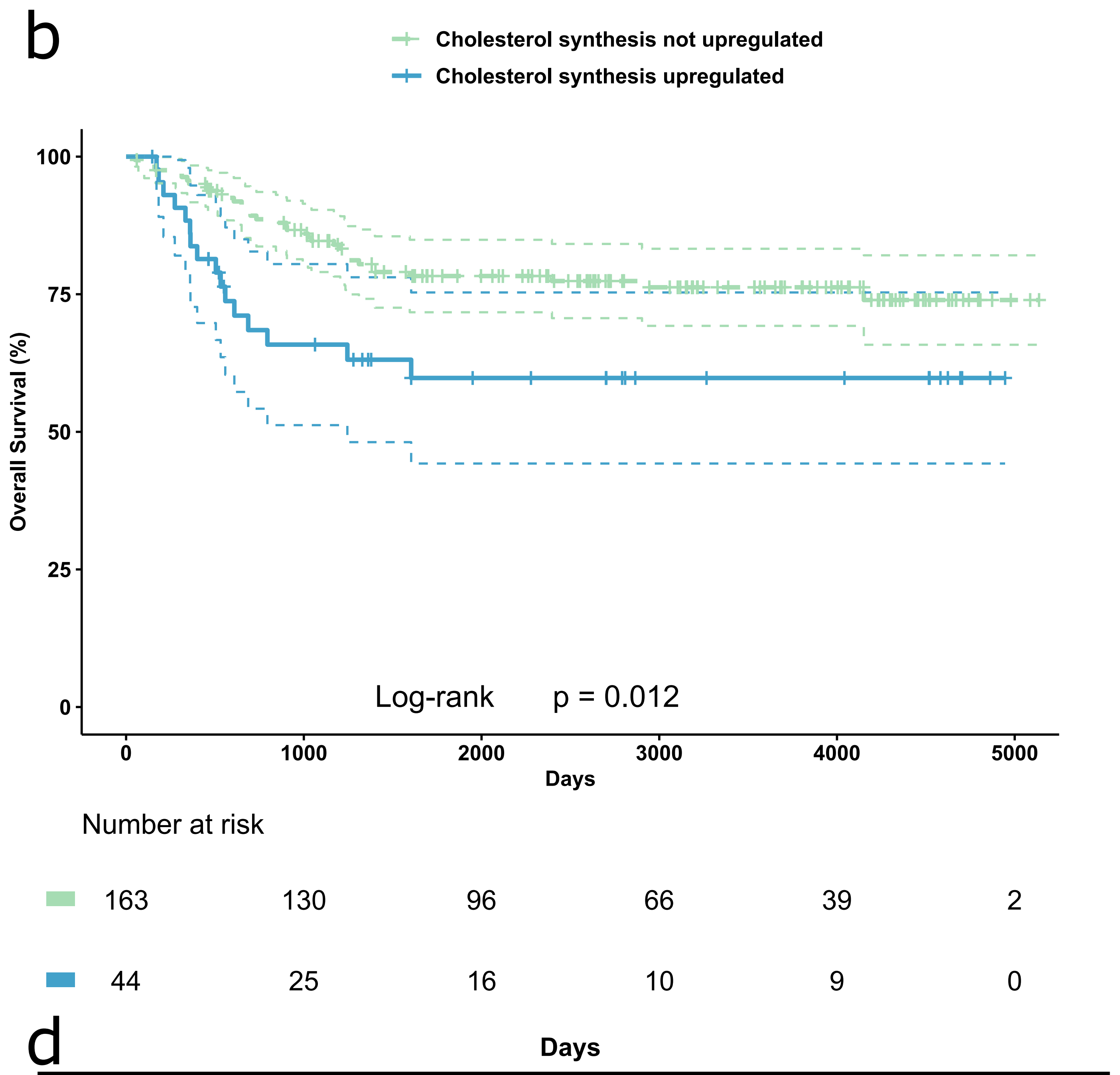

c

Risk Factor	HR (95% CI)	p
**Cholesterol synthesis upregulated**	**1.41 (0.69-2.89)**	0.3435
Day 29 MRD >0.01	3.81 (2.03-7.13)	**0.00003**
High WCC?	1.17 (0.59-2.3)	0.65678
CNS status 3	1.08 (0.52-2.26)	0.8405
High Age at diagnosis?	0.86 (0.25-2.89)	0.80303
MLL Status	0.58 (0.19-1.79)	0.34321

d

Risk Factor	HR (95% CI)	P
**Cholesterol synthesis upregulated**	**1.94 (1.04-3.63)**	**0.03834**
Day 29 MRD >0.01	3.11 (1.71-5.67)	**0.00021**
High WCC?	1.35 (0.71-2.55)	0.36427
CNS status 3	1.03 (0.53-2.0)	0.93825
High Age at diagnosis?	1.06 (0.37-3.07)	0.91603
MLL Status	0.73 (0.28-1.88)	0.50911

MASPORE

e

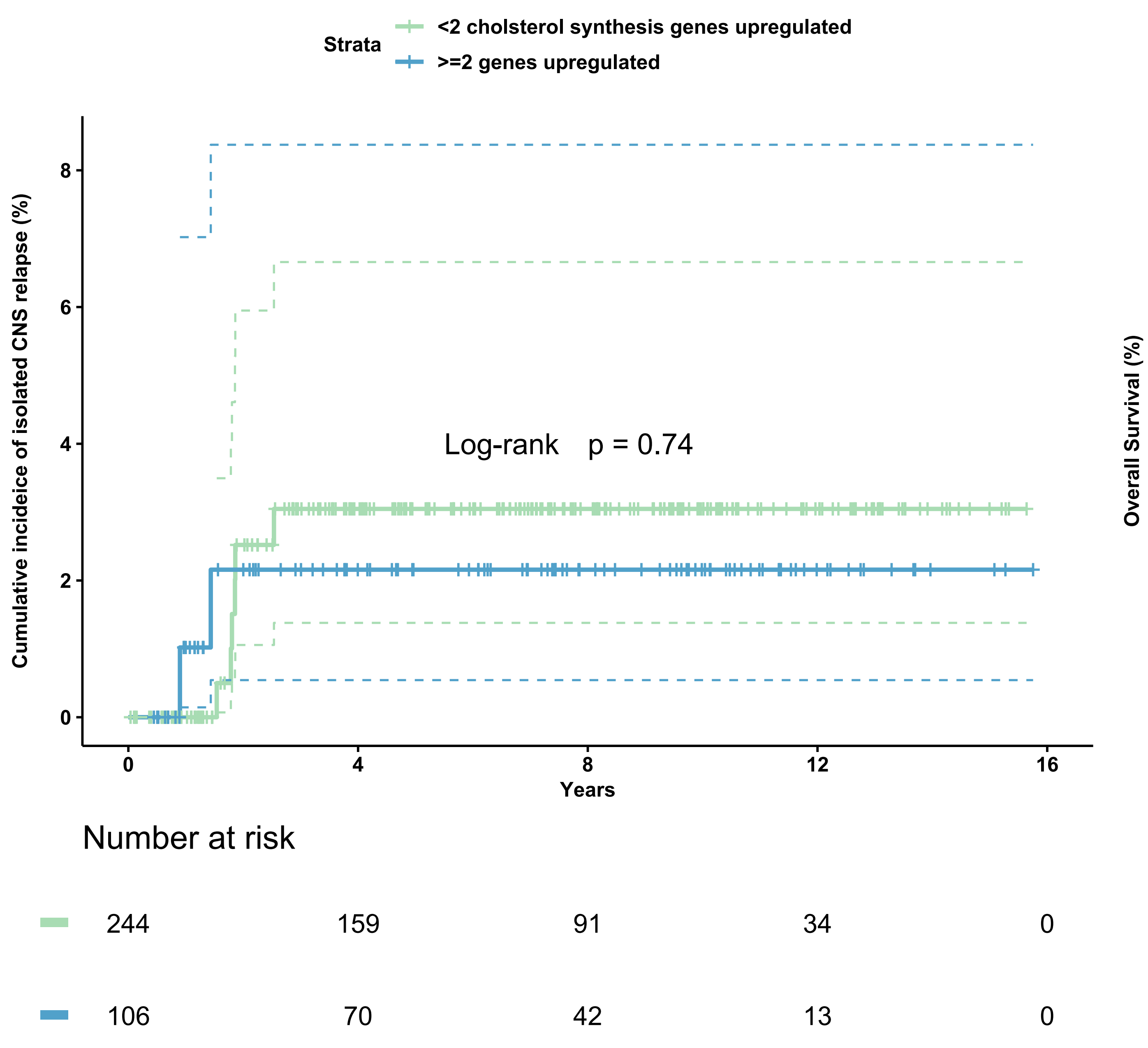

f

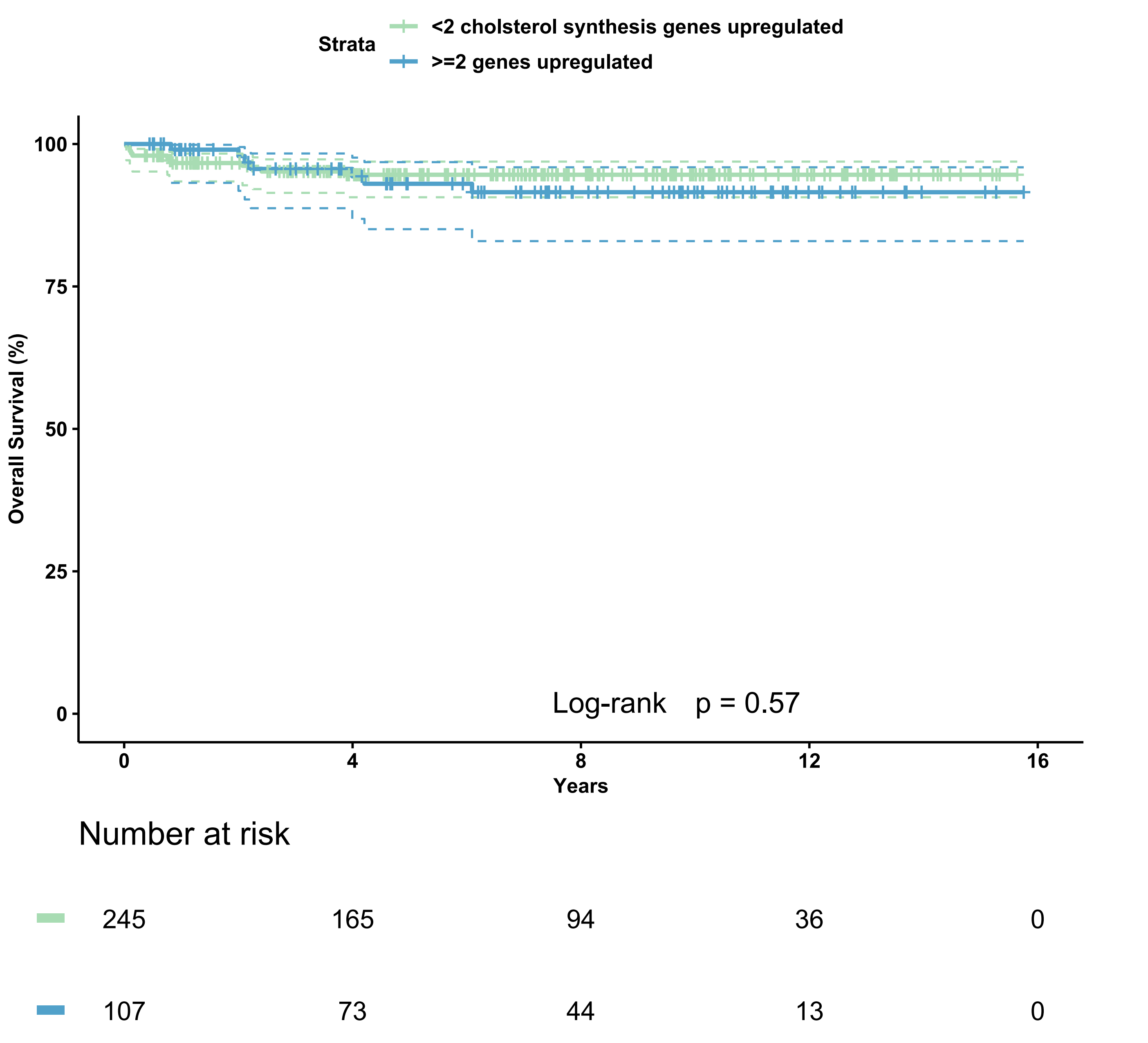

NOPHO

g

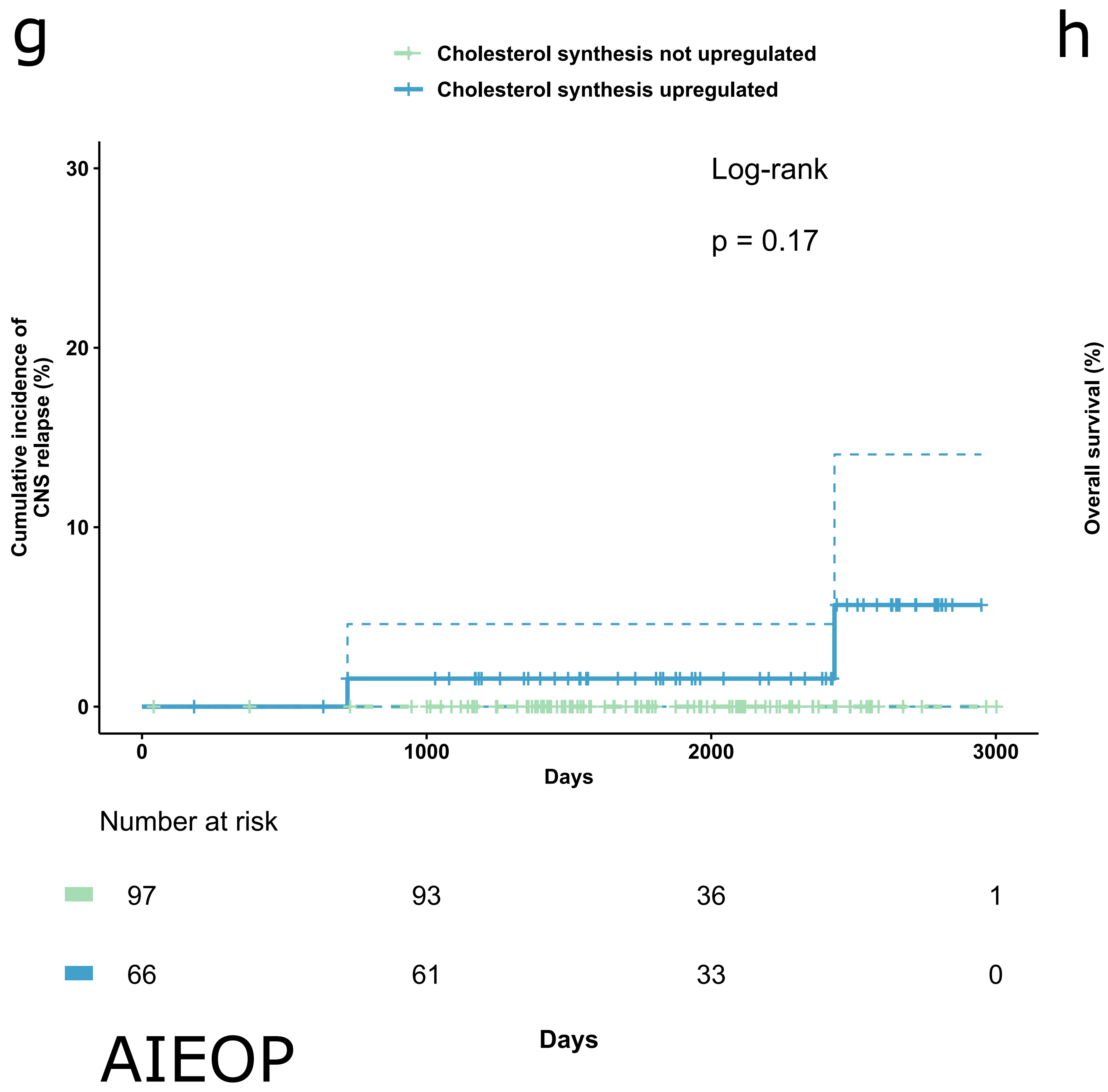

h

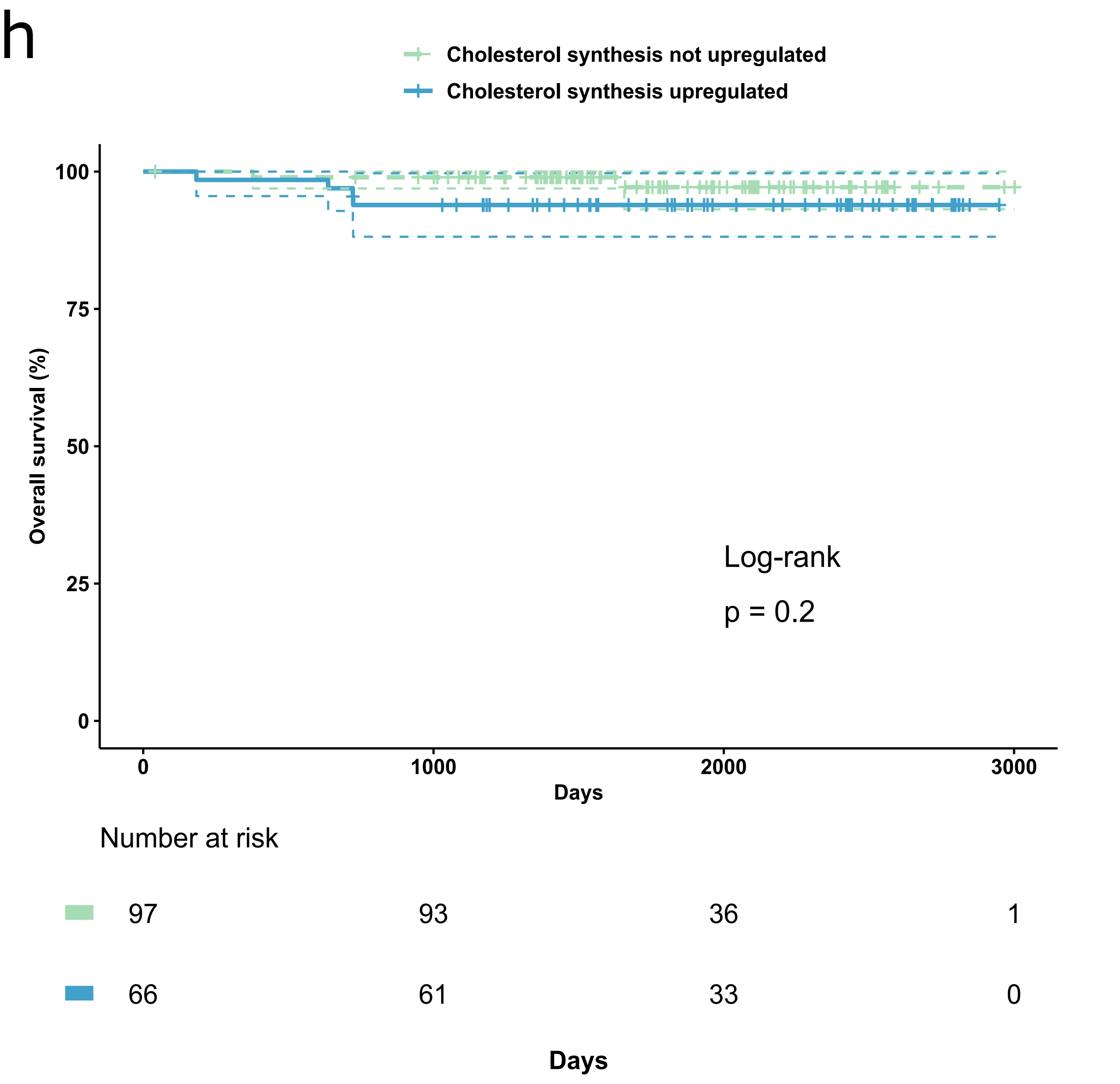

AIEOP

i

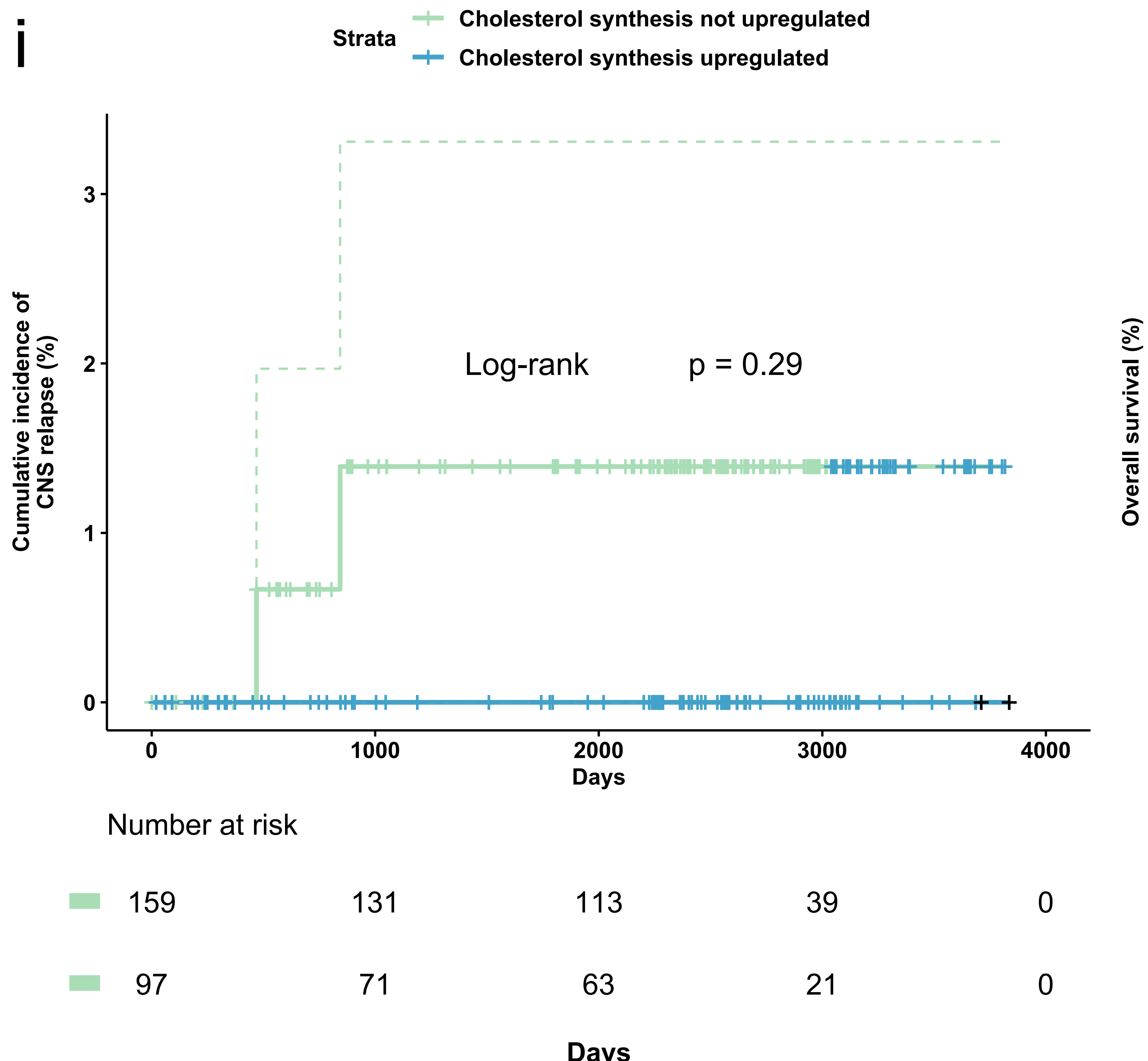

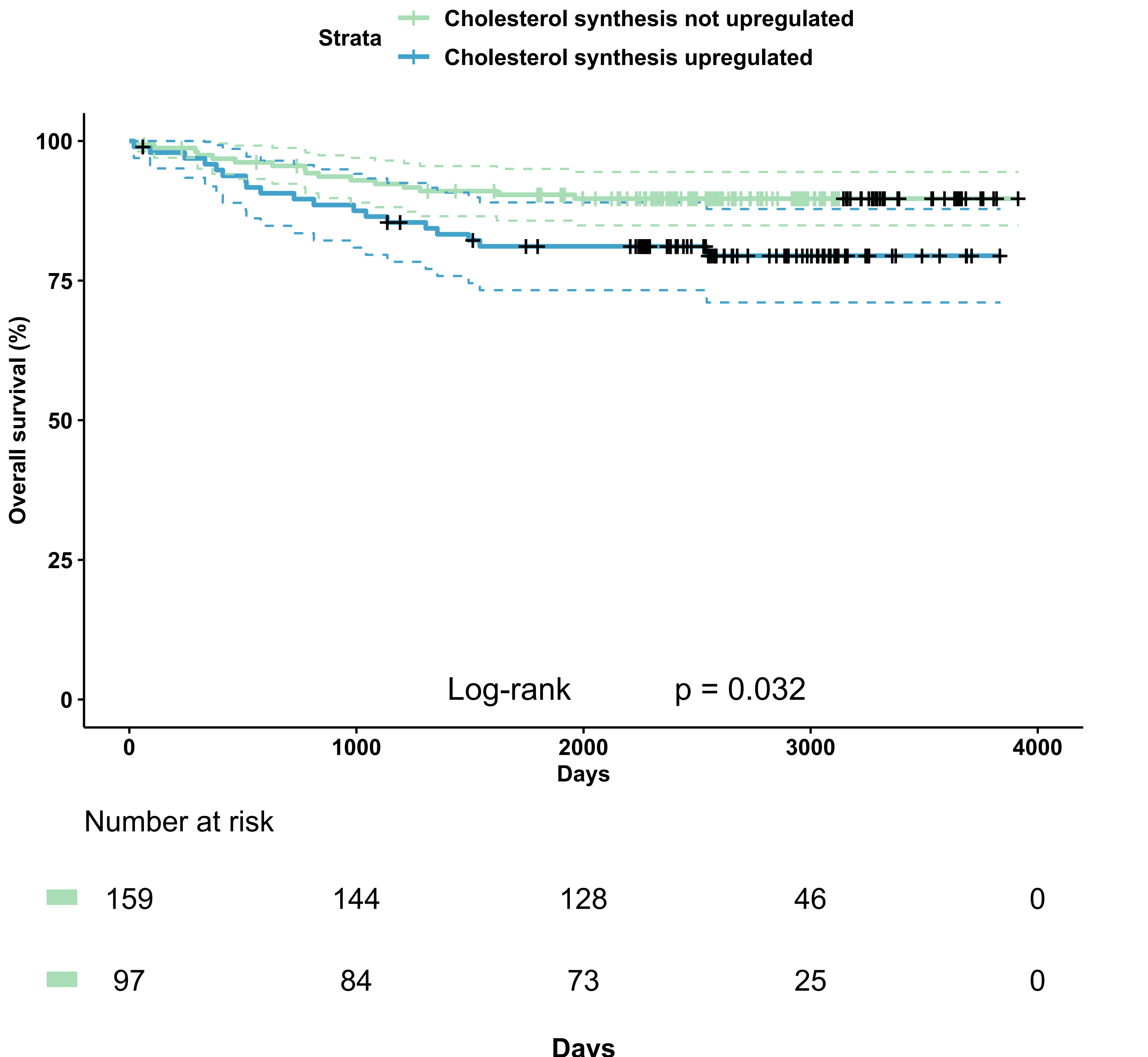

Supplement: Supplementary file 3 — Supplemental Figure 3 [file 41375_2022_1722_MOESM3_ESM.pdf]
