# Supplementary material for: Central nervous system involvement in childhood acute lymphoblastic leukemia is linked to upregulation of cholesterol biosynthetic pathways
Source: Leukemia. 2022 Oct 26;36(12):2903–7. doi: 10.1038/s41375-022-01722-x (PMC9712090; doi:10.1038/s41375-022-01722-x)
Supplement: Supplementary file 5 — Supplemental Methods [file 41375_2022_1722_MOESM5_ESM.docx]

**Supplemental Methods:**

**Human tissues:**

All human tissues obtained had written consent in accordance with Declaration of Helsinki, and were used in protocols approved by the West of Scotland Research Ethics Committee (WoSREC: 09/S0703/77). CSF samples were obtained at the Royal Hospital for Children, Glasgow (formerly Royal Hospital for Sick Children, Glasgow) and the Queen Elizabeth University Hospital and accessed via the West of Scotland CSF biobank (ALL patient samples), or the Glasgow Neuroimmunology Biobank (Control CSF). Control human plasma was obtained via the NHS Greater Glasgow and Clyde Biorepository.

**Cell lines:**

Two human ALL cell lines SEM (t(4;11) KMT2A-AFF1) and REH (t(12;21) ETV6-RUNX1) were purchased from the American Type Culture Collection (ATCC). These were maintained in tissue culture as per ATCC recommendations and were regularly monitored for mycoplasma infection. Cell line authentication was carried out annually using short tandem repeat profiling using at least 8 core STR loci.

**Xenotransplantation experiments:**

All *in vivo* experiments were carried out using UK Home Office approved protocols under licences PPL 60-4512 and PPL P6C390CF2, and in accordance with local procedures. JAX NOD.Cg-PrkdcscidIl2rgtm1Wjl/SzJ (NSG; Charles River Laboratories) mice were kept in sterile isolators with autoclaved food, bedding and water. SEM or REH cells were introduced via tail vein injection into mice between the age of 8 and 10 weeks. These cells reliably engrafted in CNS and spleen by 28 days (SEM) or 35 days (REH). At the end of the experiment (either the development of symptoms of leukaemia – weight loss or hindlimb paralysis, or day 28 (SEM) / day 35 (REH)) the mice were killed by intraperitoneal pentobarbital overdose (metabolomic experiments) or by exposure to a rising concentration of FiCO_2_.

For metabolomics experiments, CSF was obtained from mice under terminal anaesthesia, and blood drawn via the inferior vena cava or heart on confirmation of death. Samples were spun at 2000g for 15 minutes at 4°C and blood plasma obtained. Brains were carefully and rapidly removed and placed in a 2% FCS/PBS solution at 4°C, the meninges adherent to the skull were carefully and gently scraped and the scrapings suspended in solution with the brain. This solution was gently vortexed for 1 minute, and a meningeal cell suspension created by draining the solution through a 40μm filter. Spleens were rapidly removed, weighed and processed through a 40μm filter to create a cell suspension in a 2% FCS/PBS solution. Leukaemic cells were purified using the lymphoprep™ (Stemcell Technologies) centrifugation technique as per manufacturer’s instructions.

For histological analysis, mice heads were removed, stripped of soft tissues exterior to the skull and decalcified in a Hilleman and Lee 5.5% EDTA in 10% formalin solution for 2-3 weeks, then fresh EDTA for 3-4 days prior to embedding in paraffin. Paraffin blocks were then cut into 2.5μm slides, dried, and stained with Gill’s haematoxylin and Putt’s eosin.

***In-vivo* Simvastatin treatment:**

Simvasatin was chosen due to high CSF penetration compared with other statins^10^. Simvastatin (Fluorochem) was dissolved in DMSO at 250mg/ml, then diluted 1:20 in 0.5% methylcellulose and sonicated to a fine slurry. The drug was administered at a dose of 100mg/kg daily by oral gavage from day 10 post-xenotransplantation until the end of experiment at day 28 or when mice developed clinical signs of leukaemia**.**

**RNA preparation:**

RNA was extracted from purified ALL cells using Trizol™ (ThermoFisher) as per manufacturer’s instructions. The RNA was analysed using the NextSeq® 500 platform as (Illumina® Inc., CA) with poly-A mRNA selection and 75bp paired-end sequencing. Cutadapt (v1.5)^1^ was used to trim the adaptor at the 3’ end, and Sickle (v0.940, https://github.com/najoshi/sickle) was used for quality trimming using a quality threshold of 10. Reads were aligned to the reference human (Ensembl GRCh38) and murine (Ensembl GRCm38) transcriptome using k-mer statistics using Kallisto (v 0.42.4, Patcher Lab, Caltech, CA). Any reads aligning to the mouse genome were removed. In total, RNA from 65,000 genes was analysed by the lab for differential expression using DESeq2 software (v 1.13.8)^2^.

The RNA-Seq data from these xenotransplantation experiments are available via accession number [GSE135115](https://www.ncbi.nlm.nih.gov/geo/query/acc.cgi?acc=GSE135115) under the title “Gene expression profiles of MLL-AF4 and TEL-AML1 acute lymphoblastic leukaemia blasts retrieved from central nervous system and spleen”. This has two datasets, GSE135113 (“Gene expression profiles of MLL-AF4 acute lymphoblastic leukemia blasts retrieved from central nervous system and spleen”) and GSE135114 “Gene expression profiles of TEL-AML1 acute lymphoblastic leukemia blasts retrieved from central nervous system and spleen”. Results from this dataset have previously been published by our group^3^.

**Lipid Droplet analysis**

SEM cells were retrieved from the CNS and spleen of xenotransplanted NSG mice and attached to poly-L-lysine coated microscopy slides (coating was performed with 10μg/ml of poly-L-lysine (ScienCell, #0413) in clean water for 1 hour at room temperature) in PBS at 37°C for 45 min (20.000 cells per well) and fixed with 4% paraformaldehyde (BD) for 20 minutes at 37°C. Cells were stained for lipid droplets with LipidSpot™610 (Biotium, #70069) for 10 minutes at room temperature and with Hoechst 33342 (Thermo Scientific, #62249) for 10 minutes at room temperature. Vectashield Mounting Medium with DAPI was used (Vector Laboratories, H-1200). Imaging was performed with Zeiss Axio Observer 7 using x100 lens with Immersol 518 N (Carl Zeiss Microscopy GmbH) immersion oil, and images were captured with Axiocam 705 mono (Zeiss) on ZEN 3.5 software (Carl Zeiss Microscopy GmbH). The lightsource used was Colibri 5/7 (Zeiss) with 567 nm excitation filter. LipidSpot™610 was detected in the Texas Red® channel (lightsource intensity 100% at 590nm, exposure for 300.000 ms). Hoechst 33342/DAPI were detected in the DAPI channel (lightsource intensity 100% at 385nm, exposure for 150.000 ms). Image analysis was done in Fiji (ImageJ) and statistical analysis in GraphPad Prism (Version 8.0.1)

**Metabolite measurements:**

Cholesterol from CSF and plasma was measured using Amplex® Red Cholesterol Assay (ThermoFisher) according to manufacturer’s instructions.

Relative mevalonate abundance was measured using liquid-chromatography mass spectrometry (LCMS) using established protocols^4^. At 4°C 50μL of human CSF samples were mixed 1:20 in extraction solution (50% methanol/30% Acetonitrile/20% Deinoised water with a final concentration of 0.5μM 13C-labelled pyruvate, argninine, alanine, lactate and 5μM 13C Glucose). Murine CSF and plasma samples used 1μL of sample mixed 1:50 in extraction solution. Samples were thoroughly mixed in a vortex for 30 seconds, centrifuged at 16,000g for 10 minutes at 4°C, then the supernatant transferred to a glass vial and stored at -80°C until analysis.

For extraction of cellular samples, cells in microcentrifuge tubes were retrieved from storage at -80°C, and extraction solution (50% methanol/ 30% acetonitrile / 20% water as above) added to achieve a cell concentration of 1-2 x10^6^ cells/ml. Samples were mixed by pipetting to resuspend cells then agitated in a thermomixer at 1,400 rpm for 10 minutes at 4°C. Samples were then centrifuged at 16,000g for 10 minutes at 4°C, the supernatants transferred to glass vials and stored at -80°C until analysis.

Samples were analysed using a Thermo Ultimate 3000 HPLC system (ThermoFisher). 2μL of sample was loaded and run through a 150 x 2.1mm SeQuant ZIC-pHILIC column (Merck), with a preceding SeQuant 20 x 2.1mm guard column (Merck) with aqueous (20mM ammonium carbonate) and organic (acetonitrile) solvents. A gradient from 80% organic solvent/20% aqueous to 80% aqueous solvent/20% organic was run over 15 minutes, then returned to starting conditions over 7 minutes, all at 45°C. The total run time was 23 minutes.

Samples were separated by chromatography, ionised using heated electrospray ionisation (HESI) and injected into a Q-Exactive Orbitrap mass spectrometer, where ions were scanned in a range 75-1000 (m/z) with a resolution of 35,000. Polarity switching was used to produce and analyse negative and positive ions. Lock masses were used. The mass error for the spectrometer was less than 5ppm, and usually less than 2ppm. Data were acquired using XCalibur software (ThermoFisher), and analysed using TraceFinder v4.1 (ThermoFisher), and metabolites were identified by the mass of singly-charged ions and by known retention time. All metabolites detected had previously been analysed using commercial standards.

Cellular cholesterol was measured using Gas Chromatography-Mass Spectrometry (GC-MS) as described previously^5^.

All LC-MS and GC-MS was carried out by the Metabolomics Unit in the Beatson Institute for Cancer Research (BICR), Glasgow.

***In-vitro* Simvastatin treatment**

SEM cells were cultured in reduced-serum medium (1% FCS-DMEM) which has a similar cholesterol abundance to CSF (data not shown). Simvastatin (Fluorochem) was dissolved in DMSO, and diluted to 0.1% DMSO in complete media and administered at a dose of 10 µM based on dose-finding studies (data not shown). Control cells were cultured with 0.1% DMSO alone. Cholesterol-β-cyclodextran (Sigma-Aldrich) and Mevalonic Acid-Lithium Salt (Sigma) were diluted in sterile water and added at concentrations of 100µM and 20 µM respectively. Cholesterol rescue concentration was based on maximum tolerated dose assays (data not shown). Cell viability was analysed at 72 hours of culture.

**Cell viability analysis:**

Apoptosis was measured using APC-Annexin V Apoptosis Detection Kit with PI (Biolegend). Cell preparation was carried out as per manufacturer’s instructions. Briefly: cells were washed x2 in PBS, then resuspended in Annexin V Binding Buffer at a concentration of 1x10^6^ cells/ml. A 100μL aliquot was taken and 5μL APC-Annexin V and 10μL PI was added. Cells were gently vortexed then incubated in the dark at room temperature for 15 minutes. 400μL of annexin V binding buffer was added, then cells were stored at 4°C and analysed immediately by flow cytometry. Analysis was carried out using a FACScalibur (BD Biosciences) flow cytometer. Data were acquired using the FACS Diva software package and the FlowJo software package (Tree Star, Inc., Ashland, OR) was used to analyze the data.

**Bioinformatics:**

Data were obtained from the Therapeutically Applicable Research to Generate Effective Treatments (TARGET) initiative (managed by the United States’ National Cancer Institute (NCI)). The data used for this analysis are available ftp://caftpd.nci.nih.gov/pub/dcc_target/ALL/clinical/. Information about TARGET can be found at <http://ocg.cancer.gov/programs/target>).

Clinical data were available for children and young adults recruited in the P9906 trial patients between the ages 1-22 years with high-risk leukaemia. High-risk disease was defined by any of: Age- and Sex-stratified WCC (Shuster criteria), CNS 3 status, MLL gene rearrangement, or testicular involvement. Patients with Philadelphia chromosome or hypodiploidy were excluded. Patients with TEL-AML1, or trisomy 4 and 10, were excluded in the absence of CNS3 or testicular leukaemia.

These data were downloaded from the TARGET website, and microarray data from the United States’ National Centre for Biotechnology Information. Clinical file version: “TARGET_ALL_Phase_I_ClinicalData_5_8_2015_harmonized” and microarray: “GEO accession GSE11877,” data were accessed on 22/03/2016. A list of genes involved in cholesterol biosynthesis was retrieved from KEGG pathways (https://www.genome.jp) human datasets “Terpenoid Backbone Synthesis” hsa00900, and “Steroid biosynthesis” hsa00100. The gene list consisted of: CYP51A1, DHCR7, DHCR24, FDFT1, HMGCR, HMGCS1, LSS, MSMO1, MVK, LSS, SQLE, TM7SF2, PMVK, MVD, IDI1, FDPS, NSDHL, SC5D, HSD17B7, EBP, IDI2. Data for these genes were pulled from the array. Where more than 1 probe existed for a gene, the probe with highest coefficient of variation was selected. For each gene a z score was calculated for each reading using the formula:

(Ζ= (χ-μ)/σ). Data were log2-transformed prior to analysis.

All graphs, survival curves and Cox Proportional Hazards models were carried out using R packages including readxl, ggplot, rms, survminer, survival, tidyr, grid, VennDiagram, together with dependencies. Student’s t-tests were carried out using Microsoft™ Excel® 2016.

The Gene Set Enrichment Analysis v.3.0 java tool was downloaded from the Broad institute and used as per instructions using HALLMARK pathways^6^. Network analysis was performed online using the GeneMANIA platform^7^.

For data from the NOPHO patient cohort, RNA was purified from 162 diagnostic bone marrow for children and young adults (1-45 years) (147 pre-B ALL and 15 T-ALL). Geneexpression array was analyzed with Affymetrix genechip Human gene 1.0 ST. After background normalization, the RMA (Robust Multichip Average) method was used for between array normalisation and individual probe signals were summarised for individual genes (33297 core probesets). The data were analysed using the method described above.

AIEOP data were obtained from 256 paediatric B-ALL enrolled in the AIEOP ALL 200/R2006 protocols. Gene expression profile data were obtained from GeneChip HG U133 Plus 2.0 arrays (Affymetrix, Santa Clara CA, USA) and filtered for the list of genes involved in cholesterol biosynthesis used for TARGET dataset. For each gene a z-score was calculated as previously mentioned.

RNA-Seq data of patients (n=368) enrolled on MaSpore 2003/2010 studies were aligned, normalized and batch effect corrected as described previously^8^. Z-score were calculated similarly on the cholesterol biosynthesis genes using the normalized and batch effect corrected expression levels.

Further information including details of ‘R’ scripts is available if required^9^.

**References:**

1. Martin, M. (2011). Cutadapt removes adapter sequences from high-throughput sequencing reads. EMBnet.journal, 17(1), pp. 10-12. doi:https://doi.org/10.14806/ej.17.1.200

2. Love MI, Huber W, Anders S (2014). “Moderated estimation of fold change and dispersion for RNA-seq data with DESeq2.” Genome Biology, 15, 550. doi: 10.1186/s13059-014-0550-8

3. Savino, A. M., Fernandes, S. I., Olivares, O., Zemlyansky, A., Cousins, A., Markert, E. K., Barel, S., Geron, I., Frishman, L., Birger, Y., Eckert, C., Tumanov, S., MacKay, G., Kamphorst, J. J., Herzyk, P., Fernández-García, J., Abramovich, I., Mor, I., Bardini, M., Barin, E., Halsey, C. (2020). Metabolic adaptation of acute lymphoblastic leukemia to the central nervous system microenvironment is dependent on Stearoyl CoA desaturase. Nature cancer, 1(10), 998–1009. https://doi.org/10.1038/s43018-020-00115-2

4. Mackay GM, Zheng L, van den Broek NJ, Gottlieb E. Analysis of Cell Metabolism Using LC-MS and Isotope Tracers. *Methods Enzymol*. 2015;561:171-196

5. McGregor GH, Campbell AD, Fey SK, Tumanov S, Sumpton D, Blanco GR, Mackay G, Nixon C, Vazquez A, Sansom OJ, Kamphorst JJ. Targeting the Metabolic Response to Statin-Mediated Oxidative Stress Produces a Synergistic Antitumor Response. Cancer Res. 2020 Jan 15;80(2):175-188.

6. Subramanian A, Tamayo P, Mootha VK, et al. Gene set enrichment analysis: a knowledge-based approach for interpreting genome-wide expression profiles. *Proc Natl Acad Sci U S A*. 2005;102(43):15545-15550 <http://www.broad.mit.edu/gsea/>

7. GeneMania: Warde-Farley D, Donaldson SL, Comes O, et al. The GeneMANIA prediction server: biological network integration for gene prioritization and predicting gene function. Nucleic Acids Res. 2010;38(Web Server issue):W214-W220. <http://geneMANIA.org>

8. Li Z, Jiang N, Lim EH, et al. Identifying IGH disease clones for MRD monitoring in childhood B-cell acute lymphoblastic leukemia using RNA-Seq. Leukemia. 2020;34(9):2418-2429

9. Cousins AF. Investigation into survival mechanisms of malignant B cells in the central nervous system. PhD thesis, University of Glasgow 2019.

10. Saheki A, Terasaki T, Tamai I, Tsuji A. In vivo and in vitro blood-brain barrier transport of 3-hydroxy-3-methylglutaryl coenzyme A (HMG-CoA) reductase inhibitors. Pharm Res. 1994 Feb;11(2):305-11.
