# Supplementary material for: Central nervous system involvement in childhood acute lymphoblastic leukemia is linked to upregulation of cholesterol biosynthetic pathways
Source: Leukemia. 2022 Oct 26;36(12):2903–7. doi: 10.1038/s41375-022-01722-x (PMC9712090; doi:10.1038/s41375-022-01722-x)
Supplement: Supplementary file 6 — Supplemental Figure Legends [file 41375_2022_1722_MOESM6_ESM.docx]

**Supplemental Figure Legends:**

**Supplemental Figure 1:** (a) Venn diagram of differentially expressed genes in cells retrieved from the CNS and spleen of NSG mice for two separate xenotransplantation models (SEM (blue) and REH (yellow), overlap in green). (b) List of cholesterol biosynthesis genes (including related LDLR and SREBF1 and 2 genes) ranked by differential expression between leukaemia cells retrieved from CNS and spleen of NSG mice xenotransplanted with SEM and REH cells (ranked by adj. p value). (c) GeneMANIA unbiased network map, and (d) GeneMANIA top 25 gene networks (ranked by q-value), of differentially expressed genes for xenotransplanted SEM cells retrieved from the CNS vs spleen of NSG mice. (e) Geneset Enrichment Analysis (GSEA) Enrichment Plot, and (f) Top 25 “HALLMARK” pathways enriched using GSEA analysis of differentially expressed genes for xenotransplanted SEM cells retrieved from the CNS vs spleen of NSG mice.

**Supplemental Figure 2:** (a) Bar chart showing abundance of cholesterol in CSF of children at diagnosis with BCP-ALL, the same children on maintenance chemotherapy, and non-leukaemic controls. n=5 per group, p-value calculated with paired student’s t-test for “Diagnosis” vs “Maintenance Chemotherapy” groups. (b) Bar chart showing *in-vitro* effect of simvastatin on SEM cell viability after 72 hours of reduced-serum (1% FBS) culture, with mevalonate (early cholesterol precursor) rescue. n=5. (c) Bar chart as per (b) except showing effect of cholesterol rescue. n=5. (d) Plot showing effect of Simvastatin treatment on area of CNS (leptomeningeal) infiltration of xenotransplanted SEM (ALL cell line) cells. n=6 mice per group (3 male/3 female) line indicates mean/group. No statistically significant differences found between groups. (e) Example of histology slide showing CNS infiltration in NSG mice by xenotransplanted SEM cells (arrows indicate leukaemic cells in the leptomeninges). (b)/(c)/(d) P-values calculated using 2-tailed student’s t test. N.B. “n” refers to number of separate experiments throughout, with 2 technical replicates per *in-vitro* experiment.

**Supplemental figure 3:** (a) and (b) Kaplan-Meier curve showing: (a) cumulative incidence of Bone Marrow relapse, and (b) overall survival, in children with high-risk BCP-ALL by upregulation of cholesterol biosynthesis genes in the TARGET dataset. (c) and (d) Cox Proportional Hazards model of risk of: (c) Bone Marrow relapse, and (d) Overall Survival, in this dataset with traditional risk factors as defined in Fig 2 legend and upregulation of cholesterol synthesis genes. (e), (g) and (i) Kaplan-Meier curve showing cumulative incidence of isolated CNS relapse by upregulation of cholesterol synthesis in children with ALL treated on: (e)MASPORE, (g) NOPHO, and (i) AIEOP protocols. (f), (h) and (j), Kaplan-Meier curve overall survival percentage by upregulation of cholesterol synthesis in children with ALL treated on: (f) MASPORE, (h) NOPHO, and (j) AIEOP protocols.

**Supplemental Figure 4:** Schematic illustrating proposed model. Low cholesterol and lipoprotein bioavailability in the leptomeningeal microenvironment requires upregulation of genes controlling cholesterol homeostasis (shown in red). This results in preservation of many key cellular intermediates and processes required for cell survival and proliferation (shown in green). Patients whose ALL blasts express high levels of cholesterol pathway genes are at increased risk of CNS relapse.

HMGCS1=3-Hydroxy-3-Methylglutaryl-Coenzyme A Synthase 1, HMGCR=3-Hydroxy-3-Methyl-Glutaryl-Coenzyme A Reductase, SREBF1/2= Sterol Regulatory Element-Binding Protein 1/2, SQLE=Squalene Epoxidase, LDLR= Low Density Lipoprotein Receptor, MSC= Mesenchymal Stromal Cell, ALL= Acute Lymphoblastic Leukemia
